# Antibody‐mediated inhibition of EGFR reduces phosphate excretion and induces hyperphosphatemia and mild hypomagnesemia in mice

**DOI:** 10.14814/phy2.13176

**Published:** 2017-03-14

**Authors:** Bernardo Ortega, Jason M. Dey, Allison R. Gardella, Jacob Proano, Deanna Vaneerde

**Affiliations:** ^1^ Department of BiologyThe College at BrockportState University of New YorkBrockportNew York

**Keywords:** EGFR, FGF23, Klotho, magnesium deficiency, phosphate, PTH

## Abstract

Monoclonal antibody therapies targeting the EGF receptor (EGFR) frequently result in hypomagnesemia in human patients. In contrast, EGFR tyrosine kinase inhibitors do not affect Mg^2+^ balance in patients and only have a mild effect on Mg^2+^ homeostasis in rodents at elevated doses. EGF has also been shown to affect phosphate (P_i_) transport in rat and rabbit proximal convoluted tubules (PCT), but evidence from studies targeting EGFR and looking at P_i_ excretion in whole animals is still missing. Thus, the role of EGF in regulating reabsorption of Mg^2+^ and/or P_i_ in the kidney remains controversial. Here, we inject mice with the anti‐EGFR monoclonal antibody ME‐1 for 2 weeks and observe a significant increase in serum P_i_ and mild hypomagnesemia, but no changes in P_i_ or Mg^2+^ excretion. In contrast, a single injection of ME‐1 resulted in hyperphosphatemia and a significant reduction in P_i_ excretion 2 days after treatment, while no changes in serum Mg^2+^ or Mg^2+^ excretion were observed. Dietary Mg^2+^ deprivation is known to trigger a rapid Mg^2+^ conservation response in addition to hyperphosphatemia and hyperphosphaturia. Interestingly, one dose of ME‐1 did not significantly modify the response of mice to 2 days of Mg^2+^ deprivation. These data show that EGFR plays a significant role in regulating P_i_ reabsorption in the kidney PCT, but suggest only a minor role in long‐term regulation of Mg^2+^ transport in the distal convoluted tubule.

## Introduction

The kidney plays a critical role in maintaining Mg^2+^ and phosphate (P_i_) homeostasis. Within hours of Mg^2+^ deprivation, animals maintained on a Mg^2+^‐deficient diet present hypomagnesemia despite development of a rapid Mg^2+^ conservation response. These animals also show disturbed P_i_, Ca^2+^, K^+^, and Na^+^ balance (MacManus and Heaton 1969; Rude et al. 2003; Ortega et al. 2015). The discovery that a mutation in the epidermal growth factor (EGF) gene caused a rare autosomal recessive disorder known as *isolated recessive renal hypomagnesemia* (IRRH) pointed at EGF as a significant factor in the regulation of Mg^2+^ homeostasis (Groenestege et al. 2007). Although Mg^2+^ reabsorption in the kidneys takes place at the proximal convoluted tube and thick ascending limb (TAL), regulated Mg^2+^ reabsorption is mostly concentrated at the distal convoluted tubule (DCT) via the apical Mg^2+^ channel TRPM6. In the kidney, the EGF receptor (EGFR) is expressed in the TAL and DCT (Groenestege et al. 2007; Ferre et al. 2011). When expressed in HEK293 cells, the activity of TRPM6 is stimulated by addition of EGF, via activation of endogenous EGFRs (Groenestege et al. 2007). Interestingly, prolonged treatment (2 months) with cetuximab, a monoclonal antibody that targets and inhibits human EGFR, occasionally induces profound hypomagnesemia and urinary salt wasting in patients undergoing colorectal cancer treatment (Schrag et al. 2005; Groenestege et al. 2007; Izzedine et al. 2010).

Anti‐EGF‐based therapies used in the treatment of non‐small‐cell lung cancer include EGFR tyrosine kinase inhibitors such as erlotinib or gefitinib (Loriot et al. 2008), that do not result in hypomagnesemia (Altundag et al. 2005). In contrast to cetuximab, these chemical inhibitors are not species‐specific, allowing for experimental studies to be performed in non‐human models. A recent study showed that after treating mice with erlotinib for 16 days, only a mild decrease in serum Mg^2+^ was detected, while the fractional excretion of Mg^2+^ remained unchanged (Dimke et al. 2010). This study used a high dose of erlotinib (92 mg/kg/day, intraperitoneal) that resulted in serum levels of erlotinib up to 10 times greater than those found in human patients. The authors argue that monoclonal‐based therapies achieve greater bioavailability of the drug than chemical inhibitors, explaining why only a large dose of erlotinib can affect serum Mg^2+^. Another study performed in rats reported a mild decrease in plasma Mg^2+^ after 3 weeks of erlotinib treatment (10 mg/kg/day, oral), with further reduction in plasma Mg^2+^ taking place as treatment continued for up to 9 weeks (Mak et al. 2015). Thus, chemical EGFR inhibitors appear to have only moderate effects in Mg^2+^ metabolism, even when used at high doses.

Most of the filtered P_i_ is reabsorbed in the proximal kidney tubules via a transcellular process that involves P_i_ entry via apical Na‐P_i_ cotransporters, and P_i_ exit via unidentified transporters. The role of EGF in regulating P_i_ reabsorption in the proximal convoluted tubule remains controversial. In cultured LLC‐PK1 renal epithelial cells and isolated perfused rabbit PCT, EGF caused stimulation of Na‐P_i_ cotransport (Goodyer et al. 1988; Quigley et al. 1995). In contrast, EGF has been shown to inhibit NaP_i_ co‐transport activity in brush border membrane vesicles isolated from rats treated with EGF (Arar et al. 1999). The authors of this study detected a decrease in NaPi‐2 protein abundance, but there was no change in NaPi‐2 mRNA abundance. To our knowledge, no animal study has investigated the effect on P_i_ excretion of either administering recombinant EGF or blocking EGFR.

This study investigates the role of EGF in regulating P_i_ and Mg^2+^ metabolism using the rat anti‐mouse EGFR monoclonal antibody ME‐1. ME‐1 has been successfully used to block EGFR in mice, where it induces skin inflammation and triggers important histologic changes after 1 week of treatment (Surguladze et al. 2009). We first investigate the ability of long‐term (2 weeks) or short‐term (2 days) ME‐1 administration to induce hypomagnesemia or hyperphosphatemia, and then we administer a single dose of ME‐1 to Mg^2+^‐deprived or control mice in order to investigate the involvement of EGFR in the Mg^2+^ conservation response triggered by dietary Mg^2+^ deprivation.

## Materials and Methods

### Animal studies

Ten‐week‐old C57BL/6J male mice (Harlan, Madison, WI) were maintained in a temperature‐controlled room under a 12 h light‐dark cycle. For the 2‐week ME‐1 treatment, mice were maintained on standard diet (18% Protein Teklad Rodent Diet 2018, Envigo, East Millstone, NJ USA) and tap water ad libitum. ME‐1 (9.9 mg/mL in saline) was kindly provided by ImClone (Eli Lilly). Control rat IgG (Equitech‐Bio Inc, Kerrville, TX) was depyrogenated using a Detoxi‐Gel^TM^ kit (Thermo Fisher Scientific, Rockford, IL). Six doses of ME‐1 or control rat IgG (40 mg/kg) were administered intraperitoneally at regular intervals for a period of 2 weeks. On the last day of the experiment, mice were individually housed in metabolic cages, food and water consumption were measured and 24 h urine was collected under mineral oil to prevent evaporation. At the end of the experiment, mice were anesthetized using Avertin^TM^ (tribromoethanol, Thermo Fisher Scientific) and approximately 800 *μ*L of blood was collected using cardiac puncture. To produce serum, blood was allowed to clot at room temperature for 30 min and centrifuged for 20 min at 3500 g. For the experiment studying the effect of ME‐1 on the Mg^2+^‐saving response triggered by Mg^2+^ deprivation, deionized drinking water and experimental diets were provided ad libitum. Both Mg^2+^‐deficient experimental diet (0.003% Mg; Teklad TD.93106, Harlan Teklad, Madison, WI) and Mg^2+^‐control diet (0.2% Mg; Teklad TD.130120, Harlan Teklad) contained 0.45% w/w phosphorus, 0.6% w/w Ca^2+^ and 2200 IU/kg vitamin D. Prior to the experiment, all mice were maintained in Mg^2+^‐control diet for 6 days. Next, mice were injected with one dose of ME‐1 (40 mg/kg) or control IgG and allocated to their respective experimental groups and diets (Mg^2+^‐deficient or Mg^2+^‐control). Next day, mice were housed in metabolic cages, food and water consumption were measured, and 24 h urine was collected. At the end of the experiment, mice were anesthetized and serum was obtained as before. All experimental procedures were approved by the institutional animal care and use committee of The College at Brockport, State University of New York.

### Analytical procedures

All chemicals were purchased from Thermo Fisher Scientific unless otherwise stated. Total serum bilirubin was determined using a sulfanilic acid colorimetric assay (Fossati et al. 1989). A working solution was prepared containing 0.7 mol/L HCl, 2.5 mmol/L sodium nitrate, 10 mmol/L sulfanilic acid, 1 mol/L citric acid, 0.5 mol/L caffeine, 3 mol/L urea and 0.5 g/L tyloxapol. In a multiwell plate, 10 *μ*L of serum was added to 150 *μ*L of working solution and incubated for 5 min. Absorbance of samples was read at 590 nm (Synergy H1, BioTek, Ventura, CA) and the concentration in samples was calculated from a standard curve prepared using known amounts of bilirubin. Bilirubin standards were produced by dilution of a bilirubin stock solution in 5% BSA/phosphate‐buffered saline (PBS). A bilirubin stock solution was prepared by diluting 14.6 mg of bilirubin in 1 mL of 0.1 N NaOH, and then diluting 20 *μ*L of the resulting solution in 980 *μ*L of 5% BSA/PBS. Colorimetric assays for determination of Mg^2+^ (using calmagite) and inorganic phosphorus (using malachite green‐phosphomolybdate) in serum and urine, and Ca^2+^ in urine (using O‐cresolphthalein complexone) have been described before (Ortega et al. 2015). Na^+^ and K^+^ in both serum and urine were measured using a flame photometer according to the manufacturer's instructions (Jenway PFP7, Jenway Limited, Gransmore Green, Dunmow, Essex, UK). Total Ca^2+^ in serum was analyzed using flame photometry following precipitation as calcium oxalate according to the manufacturer's instructions as described before (Ortega et al. 2015).

Statistical analysis was performed using SPSS statistical software (IBM Corporation, Armonk, NY). Results from different experimental groups were compared using an independent sample *t*‐test or an ANOVA followed by Fisher's LSD test. Statistical significance was assumed at *P* < 0.05.

## Results

### Changes in the integument associated with ME‐1 administration

**Figure 1:**
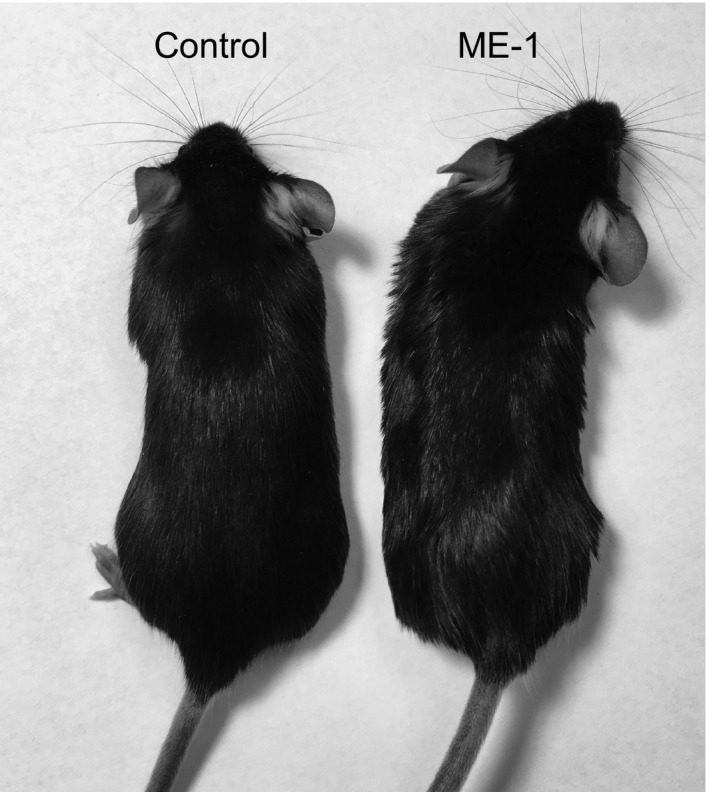
After 1 week of ME‐1 administration, C57BL/6J mice presented a disorganized and wavy hair pattern, consistent with efficient EGFR inhibition.

Patients receiving EGFR antibody therapy often experience skin rashes in areas rich in pilosebaceous units. SCID mice treated with ME‐1 display inflammatory skin changes that result in a characteristic wavy hair pattern (Surguladze et al. 2009). Consistently, C57BL/6J mice in our study showed a similar pattern from the first week of ME‐1 administration (Fig. 1), indicating that treatment was effective in inhibiting EGFR.

### Effects of long‐term ME‐1 administration on mouse electrolyte balance

After 2 weeks of ME‐1 administration, mice were placed in metabolic cages and 24‐h urine was collected. Mice were then sacrificed and serum was obtained. No significant changes in body weight or water and food intake were observed between experimental and control groups (data not shown). EGFR‐targeted anti‐tumor therapies have been shown to occasionally disrupt bilirubin metabolism and produce hepatotoxicity (Kubitz et al. 2004; Loriot et al. 2008). In our study, there were no differences in serum bilirubin between ME‐1‐treated and control mice (ME‐1: 2.62 ± 0.28 *μ*mol/L; control: 3.62 ± 0.27 *μ*mol/L, mean ± SE; *n* = 7, *P* < 0.05), showing that at the dose used, ME‐1 induced no adverse effects. As shown in Table 1, mild hypomagnesemia resulted from 2 weeks of ME‐1 administration. Furthermore, serum P_i_ in ME‐1‐treated animals increased to a level bordering hyperphosphatemia. Other electrolytes, including Ca^2+^, Na^+^, and K^+^, remained unchanged. No differences were observed in urine output or electrolyte excretion between treated and untreated animals.

**Table 1:** Serum and urine electrolyte composition of mice following a 2 week treatment with ME‐1 or control rat IgG

Measurement	Control	ME‐1	Normal range^a^
Serum			
[Mg^2+^] (mmol/L)	1.12 ± 0.01	1.04 ± 0.03^b^	1.1–1.4
[Ca^2+^] (mmol/L)	2.11 ± 0.09	2.24 ± 0.07	2.0–2.8
[P_i_] (mmol/L)	2.67 ± 0.01	2.96 ± 0.09^b^	2.2–3.0
[Na^+^] (mmol/L)	158.0 ± 2.9	155.0 ± 2.2	130–160
[K^+^] (mmol/L)	4.90 ± 0.19	5.54 ± 0.25	4.5–7.5
Urine			
Volume (mL/24 h)	1.34 ± 0.12	1.31 ± 0.17	n/a
Mg^2+^ excretion (*μ*mol/24 h)	23.65 ± 2.50	18.52 ± 3.0	n/a
Ca^2+^ excretion (*μ*mol/24 h)	0.30 ± 0.03	0.30 ± 0.03	n/a
P_i_ excretion (*μ*mol/24 h)	91.1 ± 8.5	88.0 ± 6.6	n/a
Na^+^ excretion (*μ*mol/24 h)	191.7 ± 17.5	178.8 ± 22.6	n/a
K^+^ excretion (*μ*mol/24 h)	199.3 ± 12.5	202.7 ± 17.1	n/a

a Normal serum values are based on the values provided by the Research Animal Resources at the University of Minnesota and on experiments performed in our lab.

b Data compared by unpaired Student's *t‐*test; *P* < 0.05 compared to control group. Data represent mean ± SE; *n* = 6–7.

### Involvement of EGFR in maintaining electrolyte balance during short‐term Mg_2_^+^ deprivation

**Figure 2:**
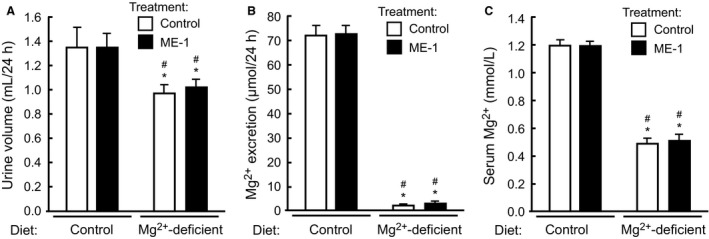
Inhibition of EGFR does not affect Mg^2+^ metabolism during dietary Mg^2+^ restriction. Mice administered ME‐1 or control rat IgG were allocated to experimental groups receiving a control diet or a Mg^2+^‐deficient diet for 2 days. At the end of the study, 24 h urine was collected using metabolic cages. (A) Mice in Mg^2+^‐deficient diet produced significantly less urine. ME‐1 did not affect urine output in either diet. (B) Mg^2+^ excretion was significantly reduced after 24 h of Mg^2+^ deprivation. Inhibition of EGFR with ME‐1 did not affect the Mg^2+^‐saving response. (C) Mg^2+^‐deprivation resulted in pronounced hypomagnesemia, which was not affected by ME‐1 administration. Data represent mean ± SE; *n* = 6–7; *P* < 0.05 versus control diet with control rat IgG (*) or control diet with ME‐1 (#). ANOVA followed by Fisher's LSD test.

**Figure 3:**
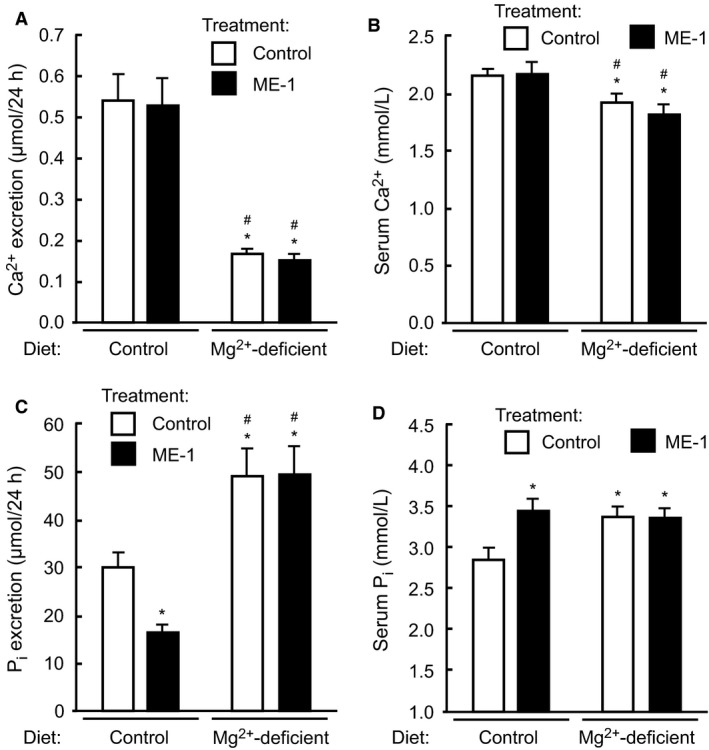
EGFR inhibition during short‐term Mg^2+^‐deprivation affects metabolism of P_i_, but not that of Ca^2+^. (A) Ca^2+^ excretion was affected by Mg^2+^ deprivation, but not by EGFR inhibition. (B) Development of hypocalcemia due to Mg^2+^ restriction was not affected by ME‐1 administration. (C) ME‐1 administration resulted in decreased P_i_ excretion in mice fed control diet. Dietary Mg^2+^ deprivation is characterized by a sharp increase in P_i_ excretion. Under these conditions, EGFR inhibition with ME‐1 was unable to decrease P_i_ excretion. (D) ME‐1 induced decreased P_i_ excretion resulted in hyperphosphatemia, similar to the effect of Mg^2+^‐deprivation. Data represent mean ± SE; *n* = 6–7; *P* < 0.05 versus control diet with control rat IgG (*) or control diet with ME‐1 (#). ANOVA followed by Fisher's LSD test.

**Figure 4:**
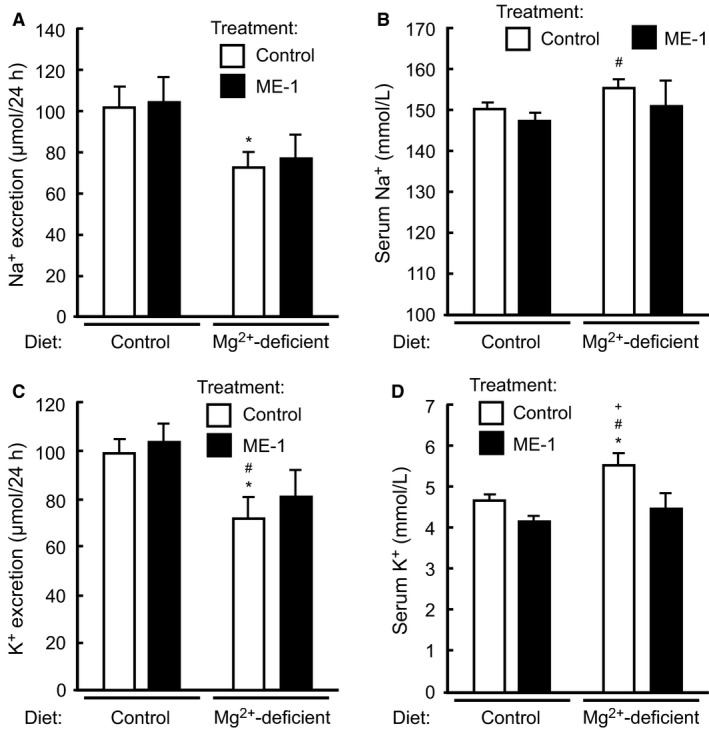
Effect of ME‐1 administration on Na^+^ and K^+^ balance. (A) Na^+^ excretion was reduced by Mg^2+^ deprivation, but not by EGFR inhibition. (B) Mg^2+^ deprivation increased serum Na^+^. (C) K^+^ excretion was reduced in Mg^2+^‐deprived mice. ME‐1 did not significantly affect K^+^ excretion in either group. (D) Serum K^+^ was increased in Mg^2+^‐deprived mice, but this effect was prevented by ME‐1 inhibition of EGFR. Data represent mean ± SE; *n* = 6–7; *P* < 0.05 versus group with control diet and control rat IgG (*), control diet with ME‐1 (#) or Mg^2+^‐deficient diet with ME‐1 (+). ANOVA followed by Fisher's LSD test.

We have previously shown that short‐term Mg^2+^ deprivation elicits a rapid Mg^2+^ saving response that results in reduced Mg^2+^ excretion, hypomagnesemia, increased serum K^+^ concentration, hypocalcemia and hyperphosphatemia (Ortega et al. 2015). Mice also decreased their urine output, and develop hypocalciuria and hyperphosphaturia. Interestingly, except hypomagnesemia, all serum perturbations are compensated after 1 week of Mg^2+^ deprivation, while excretion of Mg^2+^ and Ca^2+^ remains low, and that of P_i_ stays elevated (Ortega et al. 2015). Given that long‐term administration of ME‐1 appeared to have a modest effect on metabolism of Mg^2+^ and other electrolytes, we decided to investigate if EGFR might have instead a role in regulating the rapid Mg^2+^ conservation response observed during early hypomagnesemia. In this experiment mice were injected with a single dose of ME‐1, and then switched to Mg^2+^‐deficient diet or maintained in control diet for a day. Next, mice were placed in metabolic cages with the same diet as before for additional 24 h. As expected, Mg^2+^‐deprivation decreased urine output and Mg^2+^ excretion, in addition to inducing a profound hypomagnesemia (Fig. 2). However, treatment with ME‐1 did not affect any of these parameters, indicating that EGFR is not likely involved in Mg^2+^ conservation during the first 2 days of Mg^2+^ deprivation. As shown in Figure 3A–B, administration of ME‐1 did not modify Ca^2+^ excretion or serum Ca^2+^ concentration during normal or reduced Mg^2+^ intake. Interestingly, ME‐1 strongly reduced P_i_ excretion (Fig. 3C) and increased serum P_i_ (Fig. 3D) in mice fed control diet. As expected, Mg^2+^ deprivation decreased Mg^2+^ excretion (Fig. 2A) and increased serum P_i_ (Fig. 3D). However, ME‐1 failed to further increase serum P_i_ levels, or to curb the increase in P_i_ excretion that takes place during hypomagnesemia (Fig. 3C). Thus, EGFR is involved in the regulation of P_i_ excretion during normal conditions, but plays no additional role in regulating P_i_ during Mg^2+^ deprivation. As expected, Mg^2+^ deprivation decreased Na^+^ excretion (Fig. 4A), but ME‐1 had no effect in either Na^+^ excretion or serum Na^+^ (Fig. 4B). Furthermore, Mg^2+^ deprivation reduced K^+^ excretion and increased serum K^+^ (Fig. 4D). ME‐1 had no effect on K^+^ excretion, but interestingly, it decreased serum K^+^ during Mg^2+^ deprivation.

## Discussion

The present study investigated the effect of blocking EGFR with ME‐1 for 2 weeks and found that mice developed a moderate reduction in serum Mg^2+^, while no changes in Mg^2+^ excretion were detected. In contrast, serum P_i_ levels were clearly affected. Interestingly, a single dose of ME‐1 was effective in reducing P_i_ excretion and triggering hyperphosphatemia. Moreover, blocking EGFR with ME‐1 did not affect the acute Mg^2+^‐conservation response triggered by dietary Mg^2+^ deprivation. Taken together, our research indicates that EGF might be more involved in regulating P_i_ than Mg^2+^ metabolism in mice.

Previous studies on the ability of the EGFR inhibitor erlotinib to induce hypomagnesemia in rodents showed no effect after 10 days of treatment, and only a moderate effect (10–14%) after 3 weeks of treatment (Dimke et al. 2010; Mak et al. 2015). Chemical inhibitors do not affect Mg^2+^ metabolism in human patients, so we decided to use the rat monoclonal EGFR inhibitor ME‐1 to investigate its effects on Mg^2+^ balance in mice. Due to the possibility of mice developing an adaptive immune response to this rat monoclonal antibody, our study was limited to 2 weeks. Despite a significant effect of ME‐1 on the mice integument (Fig. 1), blocking EGFR with ME‐1 for 2 weeks resulted in a modest 7.1% decrease in serum Mg^2+^ (Table 1, *P* < 0.05). These results are in line with previous studies, thus indicating that treatment with a EGFR‐targeted monoclonal antibody has a similar effect on mice Mg^2+^ metabolism as treatment with the EGFR tyrosine kinase inhibitor erlotinib.

Within a few hours of Mg^2+^ deprivation, animals responded by rapidly reducing Mg^2+^ excretion (Shafik and Quamme 1989). In a previous study performed in mice, we showed that hypomagnesemia developed after 1 day of Mg^2+^ deprivation. Furthermore, during the first 3 days of Mg^2+^ restriction, animals increased their serum K^+^, decreased their urine output, and developed hyperphosphatemia, hypocalcemia, hyperphosphaturia and hypocalciuria. In contrast, after 1 week of Mg^2+^ deprivation, Mg^2+^ excretion was still reduced and hypocalciuria and hyperphosphaturia persisted, but serum levels of K^+^, Ca^2+^, and P_i_ have been normalized (Ortega et al. 2015). Thus, Mg^2+^ restriction elicits a quick adaptation response involving many electrolytes. In the present study, we investigated if EGF played any role in the Mg^2+^ conservation response that characterizes short‐term hypomagnesemia. As shown in Figure 2, blocking EGFR with ME‐1 did not affect serum Mg^2+^ or Mg^2+^ excretion in Mg^2+^ deprived mice. Other electrolytes, like Ca^2+^, P_i_ (Fig. 3) or Na^+^ (Fig. 4), responded to changes in the Mg^2+^ composition of the diet, but were unaffected by treatment with ME‐1. Interestingly, ME‐1 was able to prevent the increase in serum K^+^ triggered by Mg^2+^ deprivation, although we ignore the mechanism of this effect. Thus, EGF appears to play a minimal role in regulating Mg^2+^ conservation at the nephron, although, extended exposure to EGFR‐targeting medications does ultimately compromise the ability of nephrons to preserve Mg^2+^, as shown by others (Schrag et al. 2005; Dimke et al. 2010; Mak et al. 2015).

Interestingly, 2 weeks of repeated injection with ME‐1 resulted in a significant increase in serum P_i_ (table 1). To our knowledge, this is the first observation that blocking EGFR increases serum P_i_ in mice. A common cause of hyperphosphatemia is a decline in renal function. However, our study did not show a raise in serum K^+^ or a decline in serum Ca^2+^ that would suggest compromised kidney function. Previous studies in rats have shown that administration of EGF reduces expression of the Na^+^/P_i_ cotransporter NaPi‐2 in proximal convoluted tubule (PCT), potentially increasing P_i_ excretion (Arar et al. 1999). In our study, the ability of EGF to rapidly affect electrolyte transport in the nephron is demonstrated by the effect of a single dose of ME‐1 on P_i_ metabolism in mice. Two days after animals on a control diet were injected with one dose of ME‐1, P_i_ excretion was reduced by 45% (Fig. 3C) and serum P_i_ increased by 20%, resulting in hyperphosphatemia. Thus, both long‐term or short‐term administration of ME‐1 affects P_i_ metabolism. These results are in line with previous studies in rats showing that treating animals with EGF for 48 h is enough to decrease NaPi‐2 protein abundance (Arar et al. 1999). In our system, hyperphosphatemia likely results from reduction in P_i_ excretion due to increased P_i_ reabsorption at the PCT. This change is mediated by an increase in the number of NaPi‐2 cotransporters at the plasma membrane of PCT cells due to decreased EGF stimulation. A limitation of this study is that we did not analyze the effect of ME‐1 in the small intestine. In fact, hyperphosphatemia could result from increased intestinal P_i_ reabsorption, but in that case P_i_ excretion would probably be elevated.

A hypothesis for the mechanism translating EGFR inhibition into reduced NaPi‐2 expression at the PCT is provided by the recent observation that EGF increases transcription of Klotho (KL) in HEK293 cells (Choi et al. 2010). KL acts as an obligate co‐receptor of the FGF23 receptor FGFR1 that mediates inhibition of NaPi‐2 expression in PCT cells via a mechanism involving phosphorylation of Na^+^/H^+^ exchange regulatory cofactor (NHERF)‐1 (Erben and Andrukhova 2016). ME‐1‐mediated inhibition of EGFR may decrease KL expression in PCT, preventing FGF23‐mediated downregulation of NaPi‐2 expression, thus decreasing phosphaturia. In contrast, during hypomagnesemia, increased P_i_ excretion may result from a reduction of NaP_i_ cotransporters induced by high serum P_i_ (Biber et al. 1988; Markovich et al. 1995), hypoparathyroidism (Rude et al. 1978) or increased serum FGF23 (Segawa et al. 2003; van Angelen et al. 2013; Matsuzaki et al. 2013). Indeed, PTH can induce phosphorylation of NHERF‐1 and downregulation of NaPi‐2 without involvement of FGF23 or FGFR1/KL (Erben and Andrukhova 2016). However, during hypomagnesemia PTH secretion is reduced due to parathyroid gland failure (Hermans et al. 1996; Thumfart et al. 2008; Rude et al. 2009). Given that ME‐1 did not reduce P_i_ excretion in Mg^2+^‐deprived mice (Fig. 3C), the phosphaturia induced by Mg^2+^‐restriction is likely independent of both FGF23 and PTH, and might be better explained by the observed elevation in serum P_i_ (Fig. 3D).

In summary, our study shows that inhibition of EGFR with a single dose of ME‐1 had no effects on Mg^2+^ balance. Furthermore, sustained ME‐1 administration resulted in a modest effect on serum Mg^2+^ without visibly affecting Mg^2+^ excretion. In contrast, ME‐1 had a profound effect on P_i_ metabolism that became apparent from the first dose of ME‐1. Thus, in mice, EGFR appears to be more involved in the regulation of P_i_ balance than in Mg^2+^ metabolism.

## Conflict of Interest

None declared.
